# The role of “cell therapy” in osteonecrosis of the femoral head: A systematic review of the literature and meta-analysis of 7 studies

**DOI:** 10.3109/17453674.2015.1077418

**Published:** 2015-08-02

**Authors:** Costas Papakostidis, Theodoros H Tosounidis, Elena Jones, Peter V Giannoudis

**Affiliations:** ^1^Department of Trauma and Orthopaedics, G. Hatzikostas General Hospital, Ioannina, Greece; ^2^Academic Department of Trauma and Orthopaedic Surgery, University of Leeds, Leeds; ^3^Leeds Institute of Rheumatic and Musculoskeletal Medicine, University of Leeds, Leeds; ^4^NIHR Leeds Biomedical Research Unit, Chapel Allerton Hospital, Leeds, UK

## Abstract

**Background and purpose:**

The value of core decrompression for treatment of osteonecrosis of the femoral head (ONFH) is unclear. We investigated by a literature review whether implantation of autologous bone marrow aspirate, containing high concentrations of pluripotent mesenchymal stem cells, into the core decompression track would improve the clinical and radiological results compared with the classical method of core decompression alone. The primary outcomes of interest were structural failure (collapse) of the femoral head and conversion to total hip replacement (THR).

**Patients and methods:**

All randomized and non-randomized control trials comparing simple core decompression with autologous bone marrow cell implantation into the femoral head for the treatment of ONFH were considered eligible for inclusion. The methodological quality of the studies included was assessed independently by 2 reviewers using the Cochrane Collaboration tool for assessing risk of bias in randomized studies. Of 496 relevant citations identified, 7 studies formed the basis of this review.

**Results:**

The pooled estimate of effect size for structural failure of the femoral head favored the cell therapy group, as, in this treatment group, the odds of progression of the femoral head to the collapse stage were reduced by a factor of 5 compared to the CD group (odds ratio (OR) = 0.2, 95% CI: 0.08–0.6; p = 0.02). The respective summarized estimate of effect size yielded halved odds for conversion to THR in the cell therapy group compared to CD group (OR = 0.6, 95% CI: 0.3–1.02; p = 0.06).

**Interpretation:**

Our findings suggest that implantation of autologous mesenchymal stem cells (MSCs) into the core decompression track, particularly when employed at early (pre-collapse) stages of ONFH, would improve the survivorship of femoral heads and reduce the need for hip arthroplasty.

Osteonecrosis of the femoral head (ONFH) is a progressive disease caused by a critical reduction in the blood supply to the femoral head and elevation of intraosseous pressure. Although its pathogenesis is poorly understood, it is generally accepted that various traumatic and non-traumatic insults compromise the already precarious circulation of the femoral head, leading to bone marrow and osteocyte death—and eventually collapse of the necrotic segment (Mont and Hungerford 1995). It mostly affects young adults, causing considerable morbidity (Slobogean et al. 2015). The annual incidence of ONFH in the USA is estimated to be 15,000–20,000 cases (Vail and Covington 1997). Most cases without any treatment progress to femoral head collapse and joint destruction, with total hip arthroplasty being the only treatment option (Lieberman et al., 2003). Magnetic resonance imaging (MRI) has contributed to early (pre-collapse) detection of the disease, providing an opportunity for timely intervention in order to avoid femoral head collapse and subsequent joint destruction.

Various nonoperative and operative treatment modalities have been used to prevent—or at least delay—the progress of the disease towards femoral head collapse. Core decompression is a commonly used procedure, particularly in pre-collapse stages, but its effectiveness remains controversial (Ficat 1985, Learmonth et al. 1990, Markel et al. 1996, Saito et al. 1988, Yoon et al. 2001). Current research has focused on clarifying the molecular mechanisms involved in the pathogenesis of ONFH (Gangji and Hauzeur 2009, Kasten et al. 2008, Lee et al. 2009). Particular attention has been paid to multipotent mesenchymal stem cells (MSCs) and their ability to maintain mitotic multiplication while being capable of differentiating into various cellular types, such as osteoblasts, osteocytes, chondrocytes, and adipocytes (Baksh et al. 2004). Experimentally, MSCs have been shown to enhance tissue regeneration when transplanted in areas of necrotic bone (Yan et al. 2009). Various researchers have pioneered the clinical application of cell-based methods for the treatment of ONFH (Hernigou and Beaujean 2002, Gangji and Hauzeur 2005, Calori et al. 2014). Their technique was used in conjunction with the classical core decompression procedure and involved harvesting of autologous bone marrow aspirate, isolation of its mononuclear cell fraction, and injection of it into the necrotic zone of the femoral head through the canal of the preceding core decompression. This treatment strategy was based on the hypothesis that multiipotent MSCs in the bone marrow aspirate could repopulate the trabeculae of the necrotic zone within the femoral head, enhancing regeneration and remodeling of the necrotic bone (Hernigou et al. 2004).

We performed a meta-analysis to investigate whether implantation of autologous bone marrow aspirate, containing MSCs, into the core decompression track would improve the clinical and radiological results of ONFH compared to the classical method of core decompression alone. The primary outcomes of interest were structural failure (collapse) of the femoral head and conversion to total hip replacement (THR).

## Material and methods

Our systematic review of the literature adhered to the PRISMA guidelines (Liberati et al. 2009, Moher et al. 2009).

### Eligibility criteria

All full-text articles describing randomized and non-randomized control trials comparing simple core decompression with autologous bone marrow cell implantation into the femoral head for the treatment of ONFH were considered eligible for inclusion. Experimental or animal studies, case reports, editorials, letters to editors, and studies with less than10 subjects were excluded. No language restrictions were imposed.

### Literature search and data extraction

An electronic search of the MedLine database via the PubMed search machine was initially undertaken using the following terms and Boolean operators: (“osteonecrosis of femoral head” OR “avascular necrosis of femoral head”) AND (“core decompression” OR “cell therapy”) The search was further extended to the Ovid MEDLINE, CINAHL, AMED, EMBASE, Cochrane Library, and Scopus databases. The reference lists of all potentially eligible studies and review papers were carefully scrutinized for additional eligible papers. The reviewers independently assessed the titles and abstracts of the articles retrieved. For all potentially eligible articles, the full text was obtained and evaluated against the eligibility criteria. Any disagreement between reviewers was resolved by discussion. Specific demographic data, baseline characteristics, follow-up data, and outcome data were extracted from each eligible article and tabulated.

### Assessment of risk of bias (ROB) in the studies included

The methodological quality of the studies included was assessed independently by 2 reviewers (CP and TT) using the Cochrane Collaboration tool for assessing ROB in randomized studies (Higgins et al. 2011). Any discrepancy between them was resolved through discussion.

### Statistics

Binary outcomes were expressed as odds ratios (ORs) with 95% confidence intervals (CIs). Heterogeneity was assessed using Cochran chi-square test and Higgins I^2^ statistic (Cochran 1954; Higgins et al. 2003). For the former, heterogeneity was considered significant at p-values < 0.1. For the latter, an I^2^-value of greater than 50% was taken to represent significant heterogeneity. Pooling of data was performed using the Mantel-Haenszel (M-H) statistical method, with either a fixed-effects model or a random-effects model based on the degree of statistical heterogeneity present. The results of each primary study and the combined estimate of effect size are presented graphically as forest plots. Funnel plots were used to detect the presence of publication bias. RevMan 5.2 software (Review Manager, the Nordic Cochrane Center, Copenhagen, Denmark) was used to present the study findings, to produce pooled estimates of effect size, to test the presence of statistical heterogeneity, and to generate forest and funnel plots.

### Subgroup analysis

We decided a priori to explore the effect of stage of ONFH on the final outcome. Thus, we intended to separately analyze the subgroups, including hips at pre-collapse stage.

### Sensitivity analysis

Where appropriate, we planned sensitivity analyses to investigate the effect of various components of the ROB tool (such as allocation concealment, detection bias, and attrition bias) on the final outcomes.

## Results

### Search process

**Figure 1.**
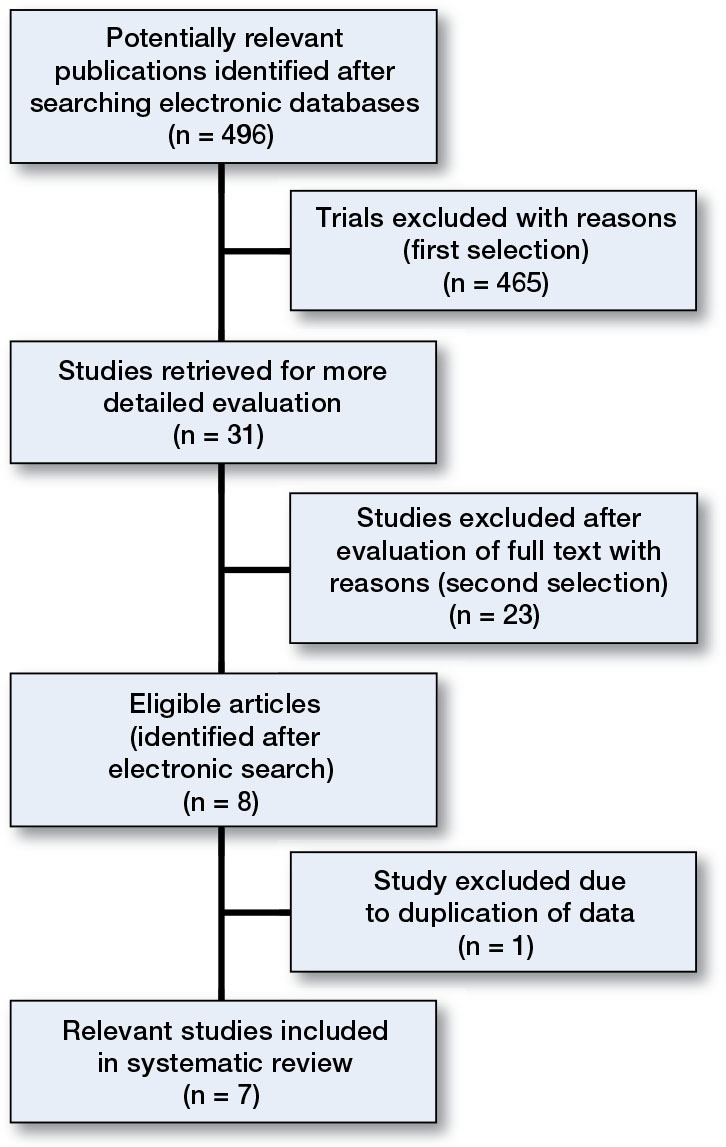
Flowchart of literature search.

Using the initial search strategy, we identified 496 citations. After application of eligibility criteria, 8 eligible reports remained for final analysis (Table and Figure 1). 1 of these reports was excluded to avoid duplication of data (Gangji et al. 2004)

**Table:** Descriptive characteristics of included studies

Reference	Yamasaki et al. 2010		Gangji et al. 2011		Sen et al. 2012		Zhao et al. 2012	
Treatment period	nr		nr		nr		May 2004 – July 2006	
Study type ^a^	Case control		Prospective control		RCT		RCT	
Compared groups	Treatment	Control	Treatment	Control	Treatment	Control	Treatment	Control
Intervention ^b^	CD + CHA + bmc	CD + CHA	CD + bmc	CD	CD + bmc	CD	CD + bmc	CD
Number of patients	22	8	10	9	40	50	50	
Mean age (SD/range)	41 (18–64)	49 (28–73)	42 (2.6)	46 (2.8)			33 (18–53)	34 (18–53)
Male / female	14:8	7:1	9:10		27:13		27:23	26:24
Number of hips	30	9	13	11	26	25	53	51
Stage of ONFH**^c^**, hips	JOA 1:2		ARCO I:2	ARCO I:2	ARCO I/II	ARCO I/II	ARCO I/II	ARCO I/II
	JOA 2:25	JOA 2:9	ARCO II:11	ARCO II:9				
	JOA 3A:3							
Precollapse/collapse, hips	27/3	9/0	13/0	11/0	26/0	25/0	53/0	51/0
Etiology of ONFH, patients (hips)								
Trauma	–	–	–	–	17 (17)		8 (8)	12 (12)
Steroids	14 (22)	2 (2)	(11)	(9)	14 (20)		10 (11)	13 (13)
Alcoholism	6 (6)	3 (4)	(1)	(1)	6 (8)		11 (11)	7 (8)
Idiopathic	2 (2)	3 (3)	(1)	(1)	1 (2)		16 (17)	13 (13)
Pregnancy	–	–	–	–	1 (2)		–	–
Cushing	–	–	–	–	1 (2)		–	–
Gaisson disease	–	–	–	–	–		5 (6)	5 (5)
Drop-outs, after initial recruitment, patients (hips)	0		4/23		(16/35)		7/100 (7/104)	
Number of followed–up, patients (hips)					(35)		50 (53)	43 (44)
Follow-up period, months	29 (19–45)		60		24		60	

**Table:** Descriptive characteristics of included studies continued

Reference	Liu et al. 2012		Lim et al. 2013		Ma et al. 2014	
Treatment period	June 2006 – Jan. 2010		Dec. 2002 – March 2004		June 2009 – Oct. 2010	
Study type	Case control		Case control		RCT	
Compared groups	Treatment	Control	Treatment	Control	Treatment	Control
Intervention ^a^	CD + bone filler (HA) + bmc	CD + bone filler (HA)	CD + bmc	CD + abg	CD + abg + bmc	CD + abg
Number of patients	17	17	86	21	21	18
Mean age (SD/range)	38 (4.9)	38 (6.1)	36 (9.7)	34 (10)	36 (8.0)	35 (11)
Male / female	13:4	14:3	69:17	16:5	15:6	13:5
Number of hips	26	27	128	31	25	24
Stage of ONFH, hips	ARCO II	ARCO II	Ficat IIa: 42	Ficat IIa: 14	Ficat I: 3	Ficat I: 4
			Ficat IIb: 37	Ficat IIb: 9	Ficat II: 17	Ficat II: 15
			Ficat III: 49	Ficat III: 8	Ficat III: 5	Ficat III: 5
Precollapse/collapse, hips	26/0	27/0	79/49	23/8	20/5	19/5
Etiology of ONFH, patients (hips)						
Steroids	(10)	(9)	48	6	(15)	(15)
Alcoholism	(15)	(14)	20	4	(4)	(3)
Idiopathic	(3)	(4)	15	10	(6)	(6)
Other	–	–	3	1	–	–
Drop-outs, after initial recruitment, patients (hips)	0/34		21 (31)/107 (159)		4 (43 initially recruited)	
Number of followed-up, patients (hips)	34 (53)		107 (159)		39 (49))	
Follow-up period, months	27 (12–40)		25 (18–32)		87 (8–134)	

**^a^**RCT: randomized control trial **^b^**CD: core decompression, CHA: calcium hydroxyapatite, bmc: bone marrow concentrate, HA: hydroxyapatite material, and abg: autologous bone grafting. **^c^**JOA: Japanese Orthopaedic Association, ARCO: Association Research Circulation Osseous

All component studies compared 2 treatment groups: (1) a group of patients suffering from ONFH who were treated with a combination of core decompression (CD) and local instillation of a bone marrow concentrate (bmc) containing osteoprogenitor cells/MSCs (the cell therapy group), and (2) a group of patients with ONFH who were treated with CD, serving as the control group. 3 of the studies were randomized control trials (RCTs) (Sen et al. 2012, Zhao et al. 2012, Ma et al. 2014), 1 was a prospective control study (Gangji et al. 2011), and the remaining 3 were retrospective case-control studies (Yamasaki et al. 2010, Lim et al. 2013, Liu et al. 2013).

### Critical appraisal and assessment of ROB in the studies included

Our systematic review of the literature was based on a lucidly stated research question, appropriately defined inclusion and exclusion criteria, a thorough search of the literature, and clearly defined outcome measures. However, almost half of the studies included were retrospective in nature (Yamasaki et al. 2010, Lim et al. 2013, Liu et al. 2013) and most of them possibly suffered from selection bias (due to lack of random generation and concealment of the allocation sequence), performance bias and detection bias (due to poor blinding of participants, personnel, or outcome assessors), and attrition bias (i.e. in most studies, follow-up losses were not taken into account in an intention-to-treat analysis) (Figure 2, see Supplementary data).

### Publication bias

Publication bias was assessed by generating funnel plots for the primary outcomes of interest (structural failure, conversion to THR). The distributions of data points in funnel plots were symmetrical, indicating avoidance of publication bias (Figure 3, see Supplementary data).

### Primary outcome measures

**Figure 4.**
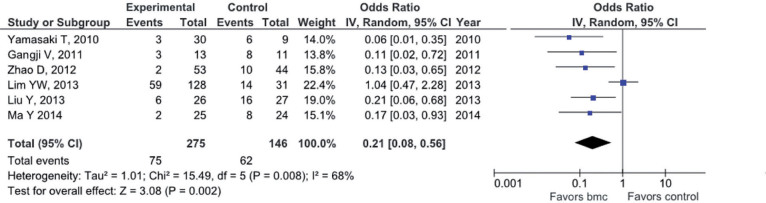
Forest plot of structural failure of the femoral head.

**Structural failure (collapse) of the femoral head (FH).** 6 studies (with 421 participants) provided relevant data (Yamasaki et al. 2010, Gangji et al. 2011, Zhao et al. 2012, Lim et al. 2013, Liu et al. 2013, Ma et al. 2014). The pooled estimate of effect size for structural failure of the FH favored the cell therapy group, as, in this treatment group, the odds of progression of the femoral head to the collapse stage were shown to be decreased by 5 times compared to the control (CD) group (OR = 0.2, 95% CI: 0.08–0.6; p = 0.02). However, this result should be interpreted with caution due to the presence of significant statistical heterogeneity (I^2^ = 68%, Q-test = 16, df = 5, and p = 0.008) (Figure 4).

**Figure 5.**
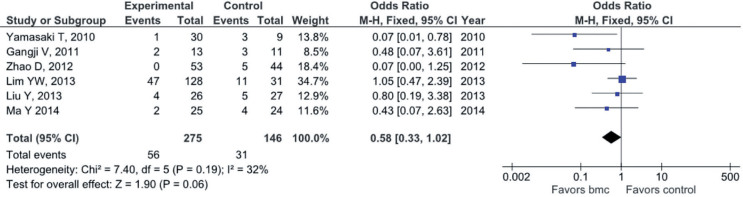
Forest plot for conversion to THR.

Conversion to THR. Relevant data were obtained from the same 6 studies (with 421 participants) (Yamasaki et al. 2010, Gangji et al. 2011, Zhao et al. 2012, Lim et al. 2013, Liu et al. 2013, Ma et al. 2014). The respective summarized estimate of effect size almost reached borderline levels of statistical significance and yielded halved odds for conversion to THR in the cell therapy group compared to the control (CD) group (OR = 0.58, 95% CI: 0.33–1.02; p = 0.06) in the absence of significant statistical heterogeneity (I^2^ = 32%, Q-test = 7.40, df = 5, and p = 0.19) (Figure 5).

### Secondary outcome measures

**Functional outcomes.** 4 studies provided relevant data, but not in a consistent way to allow us to obtain a summarized estimate of effect size of any functional outcome (Gangji et al. 2011, Sen et al. 2012, Liu et al. 2013, Ma et al. 2014).

Gandji et al. (2011) reported an overall decrease in the level of pain between the cell therapy group and the controls at 5-year follow-up.

Sen et al. (2012) found an improvement in Harris hip score (HHS) in the cell therapy group compared to the control group at 12 months postoperatively (p < 0.02). However, this difference became reduced to statistically insignificant levels at 24 months (p = 0.09).

Liu et al. (2013) found an improvement in both HHS and visual analog scale (VAS) in the cell therapy group compared to the controls at the end of follow-up (p < 0.0001 for both comparisons).

Ma et al. (2014) reported a statistically significant improvement (p < 0.001) in the cell therapy group at final follow-up compared to the baseline situation, with respect to the level of pain and joint symptoms.

### Subgroup analysis

We re-analyzed the primary outcomes of interest, including only studies reporting on early-stage (pre-collapse) ONFH.

**Structural failure of the FH (failure to collapse).** Relevant data were obtained from 5 studies (with 252 participants) (Yamasaki et al. 2010, Gangji et al. 2011, Zhao et al. 2012, Liu et al. 2013, Ma et al. 2014). The pooled estimate of effect size for structural failure of FH indicated an 8-fold decrease in the odds of collapse of the FH in the cell therapy group compared to the CD group (OR = 0.12, 95% CI: 0.06–0.26; p < 0.001) in the absence of statistical heterogeneity (Q-test = 1.81, df = 4, p = 0.77, and I^2^ = 0%) (Figure 6A, see Supplementary data).

**Conversion to THR.** 4 studies (with 208 participants) provided relevant data (Yamasaki et al. 2010, Gangji et al. 2011, Zhao et al. 2012, Liu et al. 2013). The overall estimate of effect size for conversion to THR favored the cell therapy group, although it reached only borderline significance levels (OR = 0.30, 95% CI: 0.08–1.06; p = 0.06) in the presence of a moderate degree of statistical heterogeneity (Q-test = 4.39, df = 3, p = 0.22, and I^2^ = 32%), (Figure 6B, see Supplementary data).

Some component studies of our review presented hybrid treatment protocols. In 2 of them, CD and cell therapy were combined with autologous bone graft (Lim et al. 2013, Ma et al. 2014), while in 2 other studies they had been combined with hydroxyapatite (HA) fillers (Yamasaki et al. 2010, Liu et al. 2013). After exclusion of the above studies, we created another subgroup consisting of reports comparing CD alone with CD plus application of autologous bone marrow concentrate (cell therapy) (Gangji et al. 2011, Sen et al. 2012, Zhao et al. 2012). Only 2 studies provided relevant data for the primary outcome measures (Gangji et al. 2011, Zhao et al. 2012) (Figure 7, see Supplementary data).

### Sensitivity analysis

We excluded all retrospective case-control studies, as they were considered to be more vulnerable to risk of bias, and repeated the analyses for the primary outcomes of interest, including 2 RCTs (Zhao et al. 2012, Ma et al. 2014) and one prospective cohort study (Gangji et al. 2011). This process did not produce substantially different results compared with the original ones (Figure 8, see Supplementary data).

## Discussion

Our findings indicate that the application of autologous bone marrow concentrate (autologous cell therapy) in combination with core decompression in osteonecrotic femoral heads is superior to core decompression treatment, as it was found to markedly decelerate the progression of the disease to the stage of femoral head collapse, and also limit the need for total hip arthroplasty. Some component studies also reported on clinical results, demonstrating that autologous cell therapy in addition to core decompression for the treatment of ONFH resulted in reduction of painful joint symptoms and improvement in Harris hip score compared to core decompression technique alone. However, the presentation of functional and clinical results across component studies was not consistent with producing a summarized estimate of effect size for any clinical outcome measure used, and we have had to resort to narrative reporting of these results.

Core decompression—originally described by Ficat (1985) as a method of acquiring biopsy specimens in order to establish the diagnosis of osteonecrosis—is the most widely used treatment method for ONFH at pre-collapse stage. It is generally believed that core decompression works by reducing elevated intraosseous pressure and restoring vascularity of the femoral head (Lieberman et al. 2003). However, the results of core decompression alone usually deteriorate with more advanced lesions. Thus, while CD was effective as a definitive procedure in more than 80% of cases in Steinberg stage-I disease, with more advanced stages (Steinberg stage-II and -III disease), the need for further reconstructive intervention has been documented (in 37% and 71% of cases, respectively) (McGrory et al. 2007).

Recent research has focused on the role of MSCs in the pathogenesis of osteonecrosis. Such cells were found to be reduced in number and activity in osteonecrotic femoral heads (Gangji et al. 2003). On the other hand, the capillaries within the necrotic femoral head serving as conduits for stem cell and osseous cell delivery in the bone remodeling unit are believed to be occluded by emboli or thrombosis (Jones 1985, Mont and Hungerford 1995). These findings prompted researchers to develop a new approach for the treatment of ONFH, based on implantation into the necrotic zone of the femoral head of a concentrated bone marrow preparation, containing endothelial progenitor cells (promoting angiogenesis) and MSCs (promoting osteogenesis) (Gangji et al. 2004, Hernigou and Beaujean 2002) .

The effectiveness of autologous cell therapy is highly related to the stage of the disease and also to the number of MSCs transplanted. Hernigou (Hernigou & Beaujean, 2002) showed that when patients were operated upon before collapse of the FH ensued and when they received a greater number of MSCs in the autologous bone marrow concentrate injected into the necrotic lesion, a more favorable outcome could be expected. These observations are confirmed by our findings, as the results of subgroup analysis, including those studies with early-stage disease, showed a clear superiority of autologous cell therapy over CD with a complete absence of statistical heterogeneity. In addition, the bone marrow preparations described in component studies constituted a highly concentrated autologous bone marrow aspirate containing a large number of MSCs (ranging from 1,160 per mL to 4,900 per mL) (Hernigou et al. 2009). However, the exact number of MSCs that is required to induce remodeling and repair of the osteonecrotic zone is still unknown (Gangji et al. 2011) .

### Limitations of the analysis

Our systematic review had some limitations that could have affected the validity of the results. These mainly arose from the presence of both clinical and methodological diversity across component studies, and from the fact that power calculations were not justified from the sample sizes, predisposing to occurrence of type-II error. Sources of clinical heterogeneity included age, sex, etiology of ONFH, stage of the disease, and surgical intervention. Some sources of clinical diversity (type of surgical intervention and stage of the disease) were addressed by appropriate subgroup analysis. The presence of methodological diversity predisposed to various forms of bias. The principal sources of methodological heterogeneity were biased allocation to interventions (predisposing to selection bias), poor blinding of participants, poor blinding of personnel (performance bias), poor blinding of outcome assessors (detection bias), and inappropriate handling of incomplete outcome data (attrition bias). The presence of methodological diversity was addressed with sensitivity analysis, which failed to generate substantially different results (compared to those from the initially recruited studies). We are therefore confident that our results have not been essentially distorted by the presence of methodological heterogeneity.

### Conclusion

Our findings would suggest that implantation of autologous mesenchymal stem cells into the core decompression track, particularly when employed at early (pre-collapse) stages of ONFH, would improve the survivorship of femoral heads and reduce the need for hip arthroplasty. However, better designed RCTs with adequate sample sizes, based on power calculations, are required to further determine the exact role of cytotherapy in the management of ONFH.

### Supplementary data

Figures 2, 3, 6, 7, and 8 are available at the Acta Orthopaedica website, www.actaorthop.org, identification number 8929.

## Supplementary Material

IORT_A_1077418_SM9621.pdf
