# Supplementary material for: The role of “cell therapy” in osteonecrosis of the femoral head: A systematic review of the literature and meta-analysis of 7 studies
Source: Acta Orthop. 2015 Aug 2;87(1):72–8. doi: 10.3109/17453674.2015.1077418 (PMC4940596; doi:10.3109/17453674.2015.1077418)
Supplement: IORT_A_1077418_SM9621.pdf [file IORT_A_1077418_SM9621.pdf]

Supplementary article data

The role of “cell therapy” in osteonecrosis of the femoral head

A systematic review of the literature and meta-analysis of 7 studies

Costas PAPAKOSTIDIS [1], Theodoros H TOSOUNIDIS [2,4], Elena JONES [3], and Peter V GIANNOUDIS [2,4]

[1] Department of Trauma and Orthopaedics, G. Hatzikostas General Hospital, Ioannina, Greece; [2] Academic Department of Trauma and Orthopaedic Surgery, University of Leeds, Leeds; [3] Leeds Institute of Rheumatic and Musculoskeletal Medicine, University of Leeds, Leeds; [4] NIHR Leeds Biomedical Research Unit, Chapel Allerton Hospital, Leeds, UK.
Correspondence: pgiannoudi@aol.com
Submitted 2015-05-03. Accepted 2015-06-19.

	Random sequence generation	Allocation concealment	Blinding of participants	Blinding of personnel	Blinding of outcome assessors	Incomplete outcome data	Selective reporting
Yamasaki et al. 2010	–	–	–	–	–	+	?
Gangji et al. 2011	–	–	+	–	+	–	+
Sen et al. 2012	?	–	–	–	–	?	?
Zhao et al. 2012	?	+	–	–	–	–	+
Liu et al. 2013	–	–	–	–	–	+	+
Lim et al. 2013	–	–	–	–	–	–	+
Ma et al. 2014	+	–	+	–	+	–	+

Figure 2. Risk of Bias (ROB) tool. Values are + low risk, – high risk, and ? unclear risk of bias

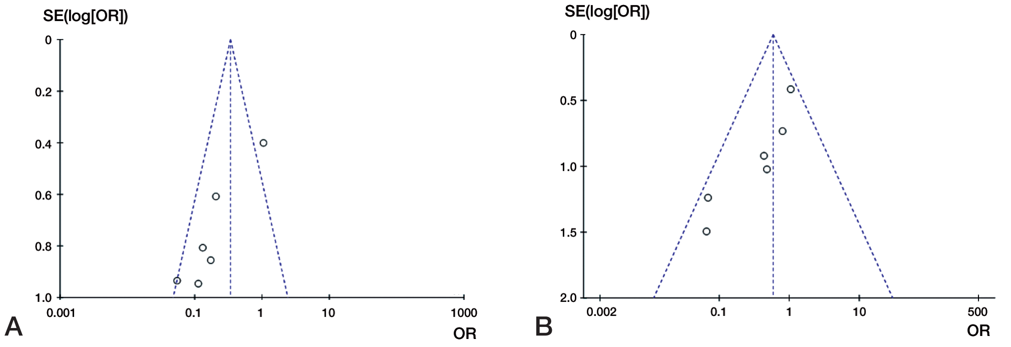

Figure 3. Funnel plots for the primary outcomes of interest. (A): Structural failure of femoral head. (B): Conversion to THR.

© 2015 The Author(s). Published by Taylor & Francis on behalf of the Nordic Orthopedic Federation. This is an Open Access article distributed under the terms of the Creative Commons Attribution-Non-Commercial License (https://creativecommons.org/licenses/by-nc/3.0)
DOI 10.3109/17453674.2015.1077418

Study or Subgroup	Cell therapy group Events	Total	Control Events	Total	Weight	Odds Ratio M-H, Fixed, 95% CI	Year
Yamasaki T, 2010	3	30	6	9	18.9%	0.06 [0.01, 0.35]	2010
Gangji V, 2011	3	13	8	11	15.1%	0.11 [0.02, 0.72]	2011
Zhao D, 2012	2	53	10	44	23.9%	0.13 [0.03, 0.65]	2012
Liu Y, 2013	6	26	16	27	27.4%	0.21 [0.06, 0.68]	2013
Ma Y 2014	0	20	6	19	14.8%	0.05 [0.00, 0.97]	2014
**Total (95% CI)**		**142**		**110**	**100.0%**	**0.12 [0.06, 0.26]**	
Total events	14		46				

Heterogeneity: Chi² = 1.81, df = 4 (P = 0.77); I² = 0%
Test for overall effect: Z = 5.54 (P < 0.00001)

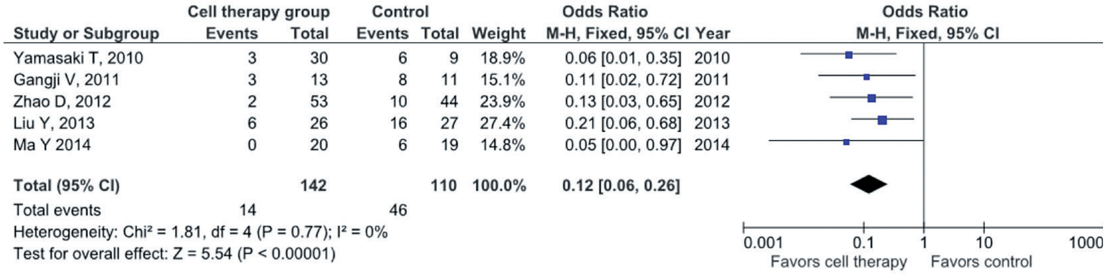

Study or Subgroup	Cell therapy group Events	Total	Control Events	Total	Weight	Odds Ratio M-H, Random, 95% CI	Year
Yamasaki T, 2010	1	30	3	9	20.3%	0.07 [0.01, 0.78]	2010
Gangji V, 2011	2	10	3	9	25.3%	0.50 [0.06, 4.00]	2011
Zhao D, 2012	0	53	5	44	15.3%	0.07 [0.00, 1.25]	2012
Liu Y, 2013	4	26	5	27	39.1%	0.80 [0.19, 3.38]	2013
**Total (95% CI)**		**119**		**89**	**100.0%**	**0.30 [0.08, 1.06]**	
Total events	7		16				

Heterogeneity: Tau² = 0.54; Chi² = 4.39, df = 3 (P = 0.22); I² = 32%
Test for overall effect: Z = 1.88 (P = 0.06)

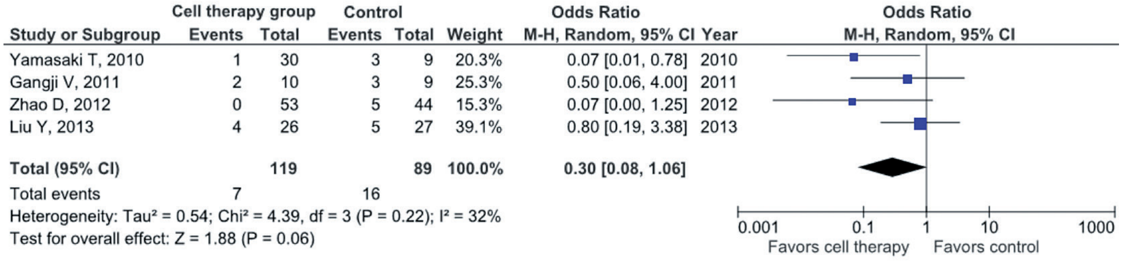

Figure 6. Subgroup analysis (pre-collapse stages). Forest plot of structural failure of femoral head (upper panel) and conversion to THR (lower panel).

Study or Subgroup	Cytotherapy group Events	Total	Control Events	Total	Weight	Odds Ratio M-H, Fixed, 95% CI	Year
Gangji V, 2011	3	13	8	11	38.8%	0.11 [0.02, 0.72]	2011
Zhao D, 2012	2	53	10	44	61.2%	0.13 [0.03, 0.65]	2012
**Total (95% CI)**		**66**		**55**	**100.0%**	**0.13 [0.04, 0.42]**	
Total events	5		18				

Heterogeneity: Chi² = 0.02, df = 1 (P = 0.89); I² = 0%
Test for overall effect: Z = 3.36 (P = 0.0008)

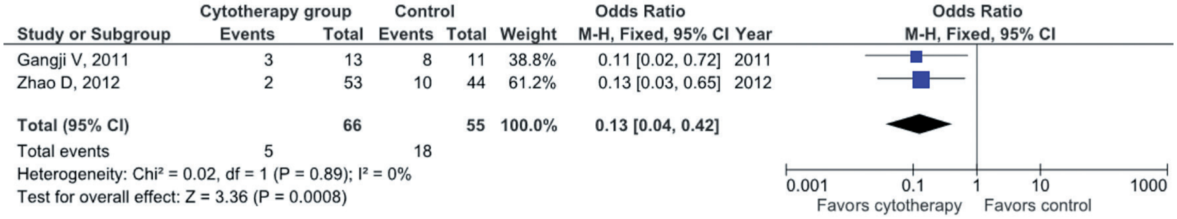

Study or Subgroup	Cytotherapy group Events	Total	Control Events	Total	Weight	Odds Ratio M-H, Random, 95% CI
Zhao D, 2012	0	53	5	44	37.3%	0.07 [0.00, 1.25]
Gangji V, 2011	2	10	3	9	62.7%	0.50 [0.06, 4.00]
**Total (95% CI)**		**63**		**53**	**100.0%**	**0.24 [0.03, 1.70]**
Total events	2		8			

Heterogeneity: Tau² = 0.49; Chi² = 1.29, df = 1 (P = 0.26); I² = 23%
Test for overall effect: Z = 1.43 (P = 0.15)

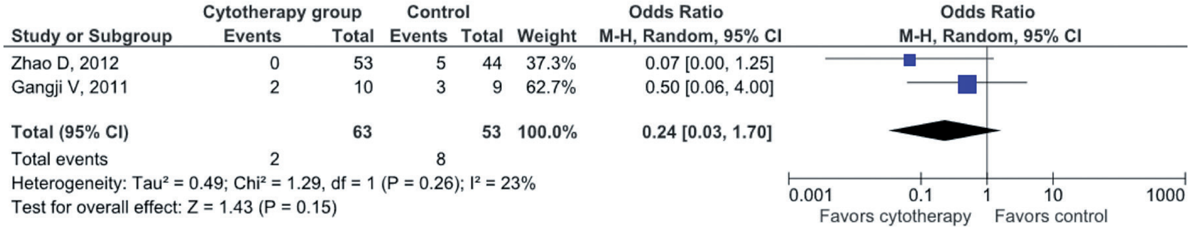

Figure 7. Subgroup analysis (cytotherapy alone versus core decompression alone). Forest plot of structural failure of femoral head (upper panel) and conversion to THR (lower panel).

Study or Subgroup	Cell therapy group Events	Cell therapy group Total	CD group Events	CD group Total	Weight	Odds Ratio M-H, Fixed, 95% CI
Gangji V, 2011	3	13	8	11	27.0%	0.11 [0.02, 0.72]
Ma Y 2014	2	25	8	24	30.4%	0.17 [0.03, 0.93]
Zhao D, 2012	2	53	10	44	42.6%	0.13 [0.03, 0.65]
**Total (95% CI)**		**91**		**79**	**100.0%**	**0.14 [0.05, 0.37]**
Total events	7		26			

Heterogeneity: Chi² = 0.12, df = 2 (P = 0.94); I² = 0%
Test for overall effect: Z = 3.93 (P < 0.0001)

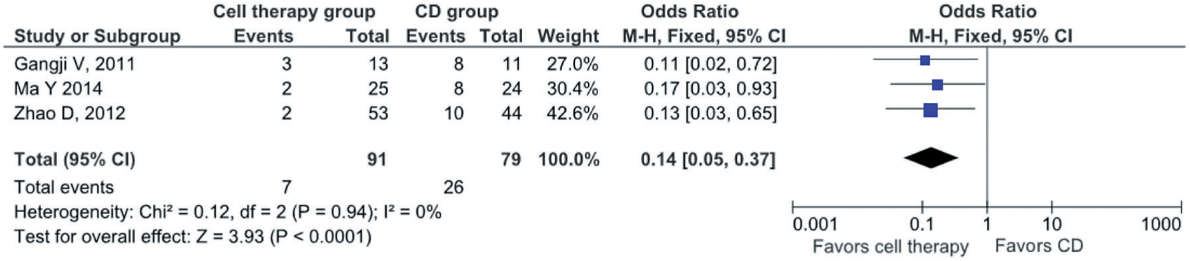

Study or Subgroup	Cell therapy group Events	Cell therapy group Total	CD group Events	CD group Total	Weight	Odds Ratio M-H, Fixed, 95% CI	Year
Gangji V, 2011	2	10	3	9	20.7%	0.50 [0.06, 4.00]	2011
Zhao D, 2012	0	53	5	44	48.6%	0.07 [0.00, 1.25]	2012
Ma Y 2014	2	25	4	24	30.7%	0.43 [0.07, 2.63]	2014
**Total (95% CI)**		**88**		**77**	**100.0%**	**0.27 [0.08, 0.86]**	
Total events	4		12				

Heterogeneity: Chi² = 1.48, df = 2 (P = 0.48); I² = 0%
Test for overall effect: Z = 2.21 (P = 0.03)

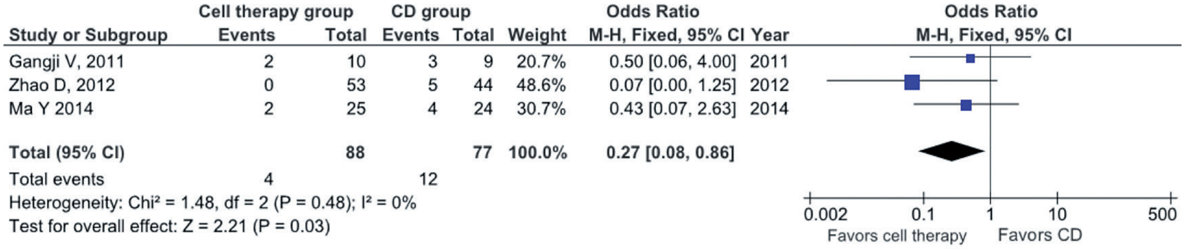

Figure 8. Sensitivity analysis. Forest plot of structural failure of femoral head (upper panel) and conversion to THR (lower panel).
